# BioBERT-powered synergy: advanced bibliometric and molecular insights into prostate cancer bone metastasis

**DOI:** 10.3389/fimmu.2025.1562559

**Published:** 2025-06-18

**Authors:** Zile Liu, Zexin Chen, Kangyi Xue, Mingkun Chen

**Affiliations:** ^1^ Department of Urology, The Fourth Affiliated Hospital of Guangzhou Medical University, Guangzhou Medical University, Guangzhou, Guangdong, China; ^2^ The First Affiliated Medical College, Southern Medical University, Guangzhou, Guangdong, China; ^3^ The Third Affiliated Medical College, Southern Medical University, Guangzhou, Guangdong, China

**Keywords:** prostate cancer, bone metastasis, bibliometric analysis, bioinformatics, BioBERT, differential gene expression, tumor microenvironment

## Abstract

**Background:**

Prostate cancer (PC) is a leading cause of male cancer mortality, with bone metastasis (BM) being a frequent and debilitating complication. Despite therapeutic advancements, the molecular mechanisms underlying BM remain poorly understood. This study aims to bridge this gap by integrating bibliometric analysis with bioinformatics to provide a comprehensive overview of the academic trends and molecular profiles associated with prostate cancer bone metastasis (PCBM).

**Methods:**

We conducted a bibliometric analysis to identify key contributors in PCBM research from 2004 to 2024 with advanced tools like BioBERT to mine gene and disease entities from the abstracts of relevant articles. Gene expression data from GSE32269 was analyzed to identify differentially expressed genes (DEGs), followed by enrichment analyses for biological functions and pathways.

**Results:**

The bibliometric review showed an increasing trend in research output, focusing on therapeutic strategies and biomarkers. Bioinformatics analysis revealed various DEGs, significantly enriched in immune response and cell adhesion pathways. Semantic relationship analysis highlighted potential shared pathways between genes and diseases, offering clues for novel immunotherapy targets.

**Conclusion:**

By integrating bibliometric analysis with bioinformatics, this study provides new insights into PCBM. Specifically, our findings emphasize the impact of reprogramming on immune cells and its role in reshaping the tumor microenvironment to support cancer cells’ evasion of immune surveillance and promotion of metastasis. These results suggest that targeting immune checkpoints and innovative combination therapies may be critical directions for improving outcomes in prostate cancer patients.

## 1 Introduction

Prostate cancer (PC) is the second most common malignancy that affects men and the fifth leading cause of mortality in men worldwide (1). PC is a complex cancer with various risk factors and the diagnosis of PC is diverse (2, 3). The treatment of prostate cancer is determined by the degree of malignancy (2–4). With its highly heterogeneous, PC can cause various degrees of metastatic spread and treatment resistance (5–7).

About 5% of men are diagnosed with metastatic prostate cancer (mPC), and 80% of them are found that the distant metastases lesions are in bone marrows (8). In spite of improvements in diagnosis and therapy for PC, bone metastasis (BM) is the main cause of death in patients with PC and there are still no effective therapy strategies up to now (9, 10).

Previous studies have suggested that prostate cancer bone metastasis (PCBM) involves complex molecular mechanisms, including genetic expression alterations (11), as well as immune system activation, cellular adhesion, and apoptotic signaling pathways (12).

Bibliometrics is a visual analysis method that creates visual representations of collaborations among authors, countries, and institutions, as well as literature, journals, and keywords (13, 14). It also helps researchers understand the academic and influential literature in a specific field. For the last few years, Bibliometrics has been increasingly employed in medical fields such as COVID-19 (15, 16), ophthalmology (17), cardiology (18), and intraoperative hypotension (19).

Despite intensive research on PCBM in recent years, there remains a notable gap in comprehensive bibliometric studies specifically focused on this field, with no prior studies systematically analyzing both research trends and molecular mechanisms. To address this, we employed bibliometric analysis tools including VOSviewer, CiteSpace, Bibliometrix, and BioBERT to analyze PCBM-related publications from 2004 to 2024. Importantly, this study represents the first integration of bibliometric analysis with bioinformatics investigation of PCBM, combining publication trend analysis with molecular pathway data from Gene Ontology (GO) and Kyoto Encyclopedia of Genes and Genomes (KEGG) databases. This dual approach provides both a macroscopic perspective on the PCBM research landscape and microscopic insights into its underlying pathological mechanisms, offering valuable guidance for future research directions.

## 2 Materials and methods

### 2.1 Data collection

We obtained all raw data from the Science Citation Index-Expanded (SCI-E) of the Web of Science Core Collection (WoSCC) database, a standardized and comprehensive resource widely used for bibliometric analysis. To ensure broad coverage of relevant literature while maintaining focus, we included all SCI-E journals in our initial search but subsequently excluded publications from clearly unrelated fields through manual screening. The data retrieval strategies are as follows: TS=(((“prostate” OR “prostates”) AND (“cancer” OR “tumor” OR “oncology” OR “neoplasm” OR “carcinoma” OR “adenocarcinoma”) AND (“osseous metastasis” OR “bone metastases” OR “bone metastasis” OR “skeletal-related events” OR “osseous metastasis” OR “skeletal metastasis” OR “skeletal complications”))). English-language articles or reviews published from 2004 to 2024 were systematically searched with the search period limited to January 1, 2004, to August 1, 2024, resulting in 5995 articles. All publications were downloaded in 1 day (August 1, 2024).

### 2.2 Data analysis and visualization

We used VOSviewer, CiteSpace, Bibliometrix, and Microsoft Excel 2021 for visual analysis. The publications, countries/regions, institutions, authors, journals, citations, keywords, and references were obtained from the WOSCC in text format files. To ensure accuracy and consistency, we standardized country names, author names and closely related keywords using VOSviewer’s built-in thesaurus for automated normalization, followed by manual verification of atypical cases (e.g., “P.R. China”), which significantly improved data uniformity while reducing manual effort. VOSviewer, CiteSpace, and Bibliometrix have been widely utilized for bibliometric analysis in recent literature. With VOSviewer, we extracted raw data and visually analyzed the distribution, collaboration, and trends of countries and institutions as well as networks of authors, citations, and keywords. CiteSpace was used to visually analyze keywords, journals, the keyword timeline, keyword bursts and reference bursts from the above-extracted data. On Bibliometrix, we analyzed countries, institutions, journals, literatures, and keywords with factorial map, trend topic figure, and so on. As for Microsoft Excel 2021, we use it for data statistics as well as creating tables and charts.

To further investigate the pathways related to the tumor microenvironment in PCBM, KEGG pathway and GO enrichment analyses were conducted. Expression profile dataset GSE32269, containing 29 BM bone marrow samples and 4 normal bone marrow samples, was downloaded from the GEO database for further analysis. The gene expression dataset GSE32269 was selected for its unique focus on bone metastasis-specific molecular profiles, containing 29 bone metastasis (BM) samples and 4 normal bone marrow controls—a rare comparative design that enables direct analysis of metastatic bone lesions. While newer datasets provide larger cohorts, they primarily focus on primary prostate tumors and lack matched metastatic bone tissue. Significantly expressed genes were screened from these samples. Before analysis, all raw data were reprocessed, mapping probes to gene symbols. The reprocessed data were normalized using the limma package in R (20). Adjusted p-values less than 0.05 and |logFC| > 2 were set as the screening thresholds. This stringent cutoff was selected to prioritize genes with substantial biological relevance to bone metastasis while minimizing false positives inherent in bulk RNA-seq datasets. The |logFC| > 2 criterion aligns with prior studies focusing on PCBM, ensuring consistency in cross-study comparisons. Subsequently, enrichment analysis was performed using the clusterProfiler package in R (21), with a false discovery rate (FDR) < 0.05 set as the threshold for identifying significantly enriched terms.

In BioBERT, large-scale biomedical datasets are used to train a domain-specific language representation model. As a pretraining step, we initialized BioBERT with BERT weights and trained it on public domain corpus (English Wikipedia and BooksCorpus). The collected data was then mined using this optimized model (22).

**Figure 1:**
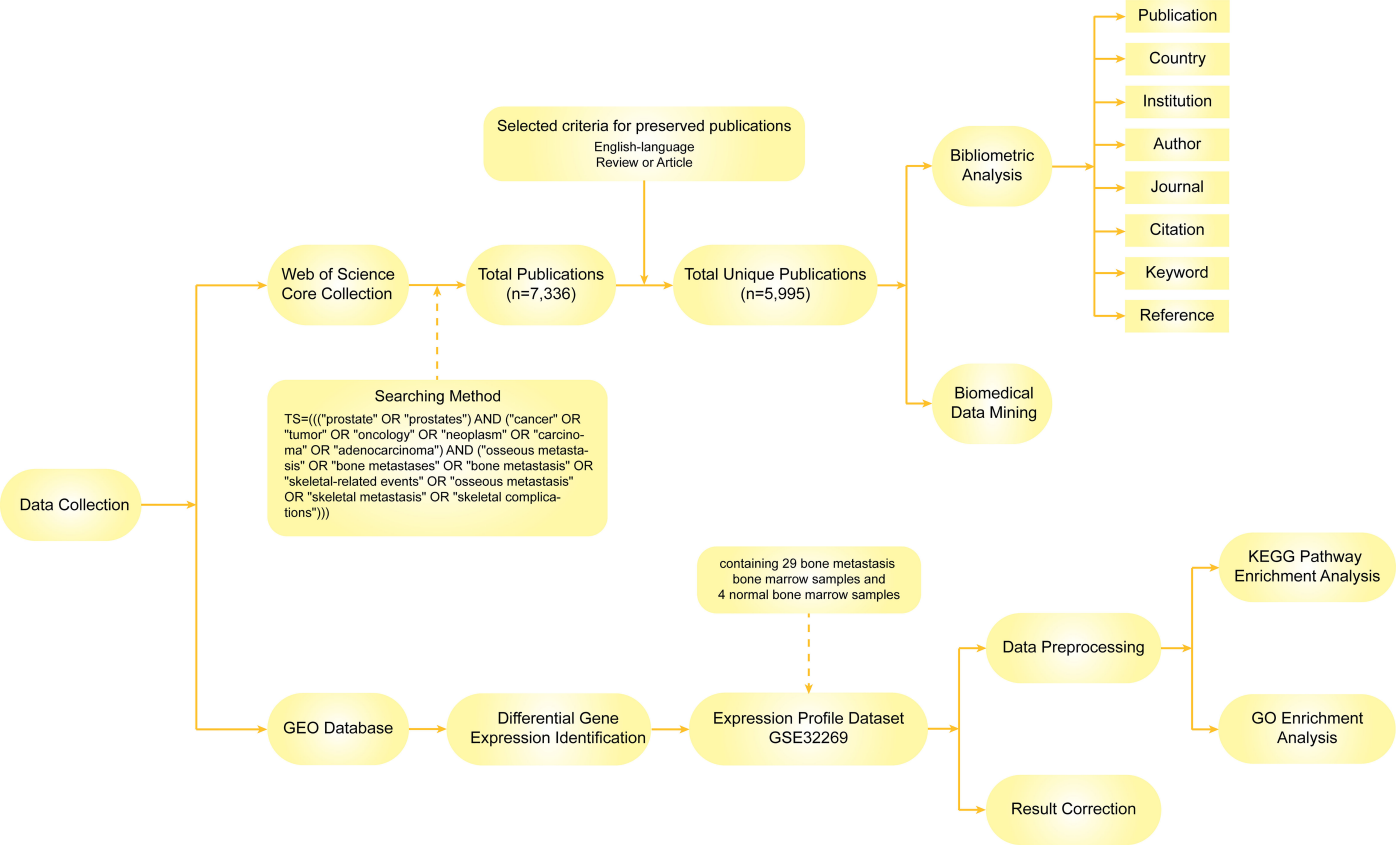
The workflow of data identification and selection.

**Figure 1** illustrates the process of data identification and selection strategies in this study.

## 3 Result

5,995 publications utilized in this study were authored by 30,091 individuals from 2,732 institutions across 95 countries and were published in 262 journals.

### 3.1 Annual publications and citations

**Figure 2:**
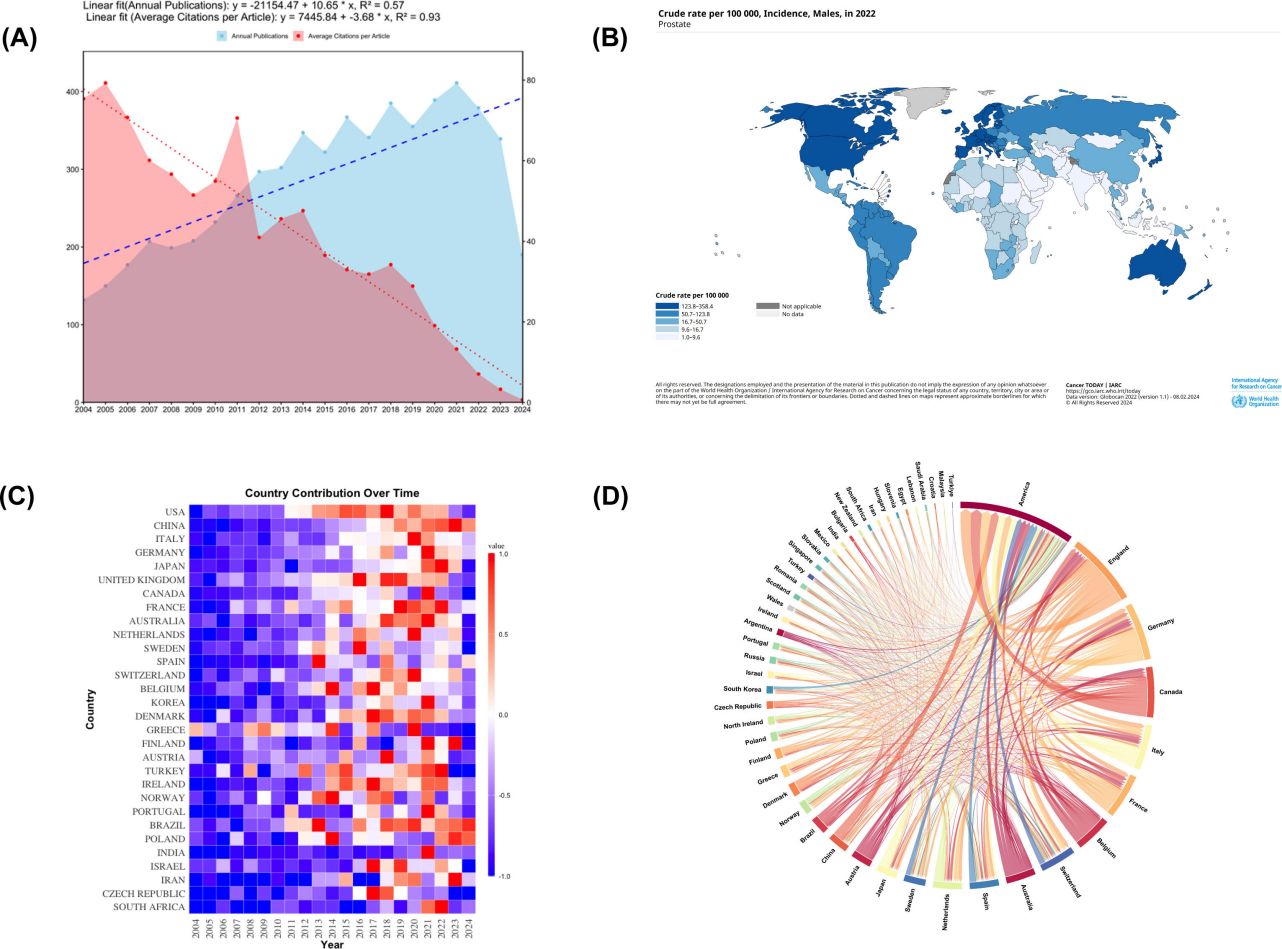
**(A)** Description of publication volume and citation frequency. From 2004 to 2024, the annual publication volume in the field of prostate cancer bone metastasis shows a continuous growth trend (linear fit equation: y = -21154.47 + 10.65 * x, R² = 0.57), while the average number of citations per article tends to decrease (linear fit equation: y = 7445.84 - 3.68 * x, R² = 0.93). **(B)** The global incidence rate of prostate cancer. The figure was downloaded from https://gco.iarc.who.int/today/en/dataviz/maps-heatmap?mode=population&sexes=1&cancers=27&key=crude_rate. **(C)** A heatmap of countries involved in research on prostate cancer bone metastasis. **(D)** Distribution and international cooperation of countries/regions. The thickness of the lines indicates the frequency of collaboration, with thicker lines representing stronger partnerships.

The analysis of publication trends over the past two decades shows a general upward trajectory in PCBM research output, with notable fluctuations. The peak publication year was 2021 (n=411), while the lowest output occurred in 2004 (n=132). **Figure 2A** demonstrates the trend of annual publication volume and average citations per article in PCBM research from 2004 to 2024. It indicates that while research output is increasing, the academic influence of each article is diminishing.

Complementing these bibliometric findings, **Figure 2B** presents epidemiological data on the global incidence of PC, with more detailed country-specific rates available in **Supplementary File 1**.

### 3.2 Distributions of countries/regions

Geographical analysis reveals distinct patterns in research productivity and collaboration. The USA emerges as the dominant force in PCBM research, leading in both publication output and citation frequency (**Table 1**). **Supplementary Figure 1A** provides a visual representation of global publication distribution, highlighting regional research concentrations.

**Table 1:** Top 10 countries/regions related to prostate cancer bone metastasis research.

Rank	Country	Document	Rank	Country	Total Link Strength	Rank	Country	Citation
1	USA	2,239	1	USA	1,902	1	USA	118,430
2	China	845	2	England	1,163	2	England	38,952
3	England	559	3	Germany	923	3	Germany	26,473
4	Germany	540	4	Canada	802	4	Canada	25,568
5	Italy	529	5	Italy	721	5	Italy	21,694
6	Japan	493	6	France	714	6	France	21,106
7	Canada	440	7	Belgium	582	7	Australia	17,978
8	France	287	8	Switzerland	567	8	China	17,494
9	Australia	239	9	Australia	470	9	Japan	14,848
10	Switzerland	219	10	Spain	466	10	Netherlands	11,870

The temporal evolution of national contributions is illustrated in **Figure 2C**, showing how different countries have entered and contributed to the field over time. **Figure 2D** further details the collaborative network among countries/regions. **Figure 2D** further elucidates these patterns by mapping international collaboration networks, revealing how knowledge flows between nations. These networks are categorized into four distinct clusters in **Supplementary Figure 1B**, where color coding indicates collaborative relationships.

A historical trend analysis reveals a distinct globalization pattern in PCBM studies over time. Early foundational work was predominantly led by research teams from the USA, Canada, Greece, and Wales, as shown in **Supplementary Figure 1C**. More recently, this research landscape has expanded significantly, with China, Iran, and Singapore emerging as major contributors.

At the institutional level, analysis of research productivity shows clear leaders in the field. Among the top 10 institutions by publication output (**Table 2**), seven are based in the USA, two in England, and one in Canada, with the University of Michigan producing the highest number of articles (n=164). **Table 2** also highlights institutions with strong total link strength and a large number of citations, mostly in the USA. When considering total institutional output including affiliated organizations, the University of Texas System leads with 575 publications (**Supplementary Figure 1D**).

**Table 2:** Top 10 institutions related to prostate cancer bone metastasis research.

Rank	Institution	Citation	Original country/region	Rank	Institution	Publication	Original country/region	Rank	Institution	Total link strength	Original country/region
1	Univ Michigan	11,479	USA	1	Univ Michigan	164	USA	1	Univ Washington	349	USA
2	Amgen Inc	9,486	USA	2	Univ Texas Md Anderson Canc Ctr	162	USA	2	Mem Sloan Kettering Canc Ctr	329	USA
3	Massachusetts Gen Hosp	8,914	USA	3	Univ Washington	124	USA	3	Inst Canc Res	305	England
4	Mem Sloan Kettering Canc Ctr	8,587	USA	4	Mem Sloan Kettering Canc Ctr	123	USA	4	Univ Texas Md Anderson Canc Ctr	273	USA
5	Univ Texas Md Anderson Canc Ctr	8,191	USA	5	Amgen Inc	103	USA	5	Massachusetts Gen Hosp	243	USA
6	Univ Washington	8,084	USA	6	Univ Sheffield	100	England	6	Univ Michigan	238	USA
7	Univ Montreal	7,358	Canada	7	Inst Canc Res	96	England	7	Duke Univ	236	USA
8	Univ Sheffield	6,825	England	8	Univ Toronto	85	Canada	8	Univ Calif San Francisco	231	USA
9	Johns Hopkins Univ	6,761	USA	9	Johns Hopkins Univ	82	USA	9	Univ Sheffield	224	England
10	Inst Canc Res	6,596	England	10	Univ Calif San Francisco	79	USA	10	Amgen Inc	212	USA

**Figure 3:**
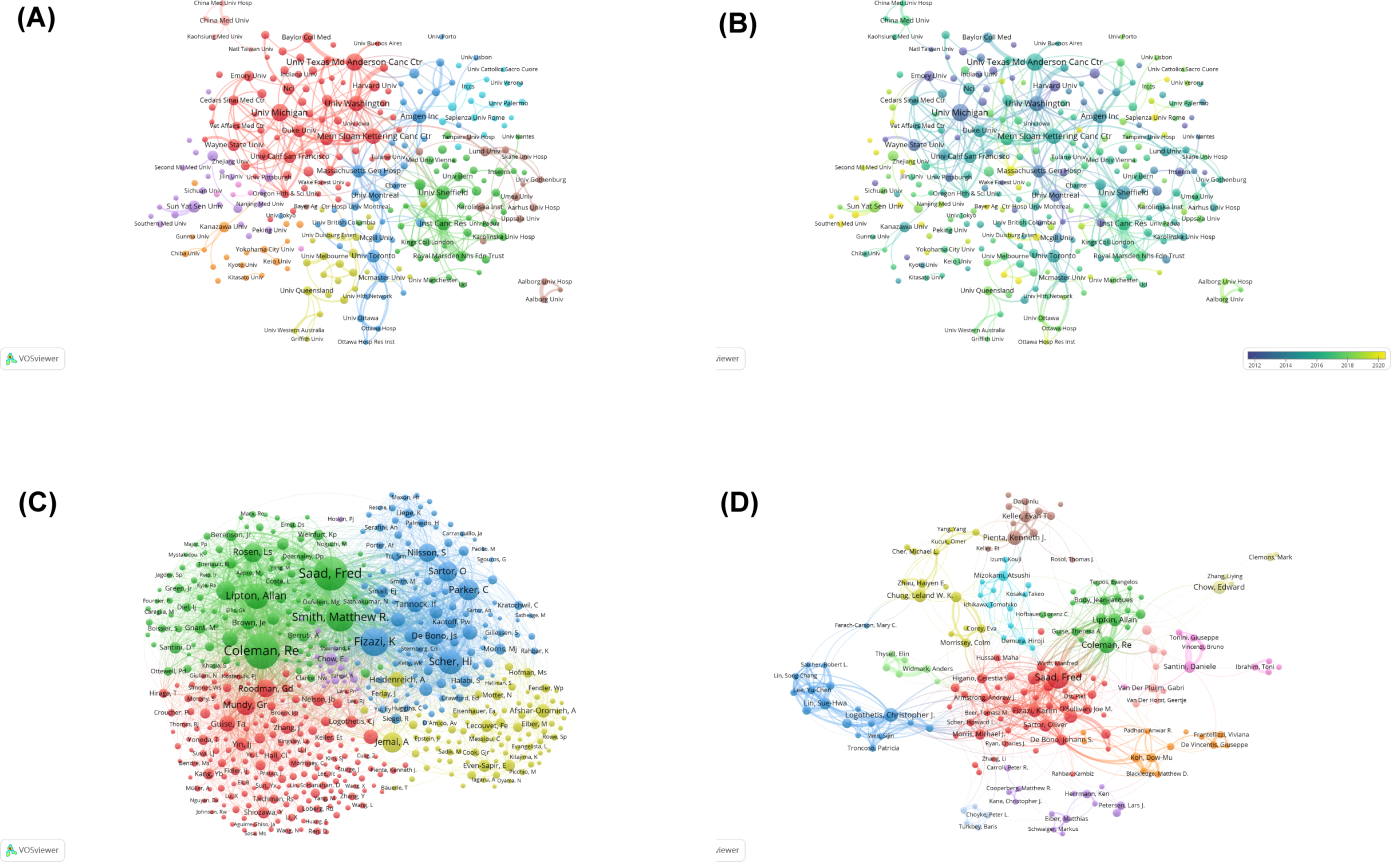
**(A)** Visual cluster analysis of the collaborative network of institutions. Different nodes represent different institutions, with various colors indicating clusters that share different affinities. The size of the nodes is determined by the publication output of the institutions. **(B)** Overlay map of historical trends in the number of articles published by each affiliation. The purple nodes represent countries that have studied this field earlier, whereas the yellow nodes indicate countries that have participated more recently. **(C)** Visual analysis of co-cited authorship network. The nodes indicate authors, and the size of nodes represents the frequency of their occurrence. **(D)** Visual analysis of collaborations among authors. Different nodes represent different authors; the lines between nodes indicate a cooperative relationship, and the thicker the lines are, the closer the relationship is; the size of nodes indicates the number of published documents, and the larger the node is, the more the number of publications.

**Figure 3A** illustrates the cooperation among institutions, organized into nine distinct clusters. The historical publication trends of individual institutions (**Figure 3B**) demonstrate particularly rapid growth in research output from Chinese institutions in recent years, including Sun Yat-sen University, Southern Medical University, and Nanjing Medical University.

### 3.3 Distributions of authors and co-cited authors

The 30,091 authors contributing to PCBM research exhibit a characteristic productivity distribution. Saad Fred stands out as both the most prolific and influential author in the field (**Table 3**, **Supplementary Table 1**).

**Table 3:** Top 10 authors in the field of prostate cancer bone metastasis.

Rank	Author	Document	Country/region	Institution	Rank	Author	Citation	Country/region	Institution
1	Saad, Fred	109	Canada	Univ Montreal	1	Saad, Fred	9,228	Canada	Univ Montreal
2	Coleman, RE	68	England	Univ Sheffield	2	Coleman, RE	8,952	England	Univ Sheffield
3	Pienta, Kenneth J.	51	USA	Johns Hopkins Univ	3	Fizazi, Karim	5,918	France	Univ Paris Saclay
4	Logothetis, Christopher J.	48	USA	Univ Texas MD Anderson Canc Ctr	4	Lipton, Allan	5,750	USA	Penn State Univ
5	Chow, Edward	47	Canada	Univ Toronto	5	Smith, Matthew R.	5,345	USA	Massachusetts Gen Hosp
6	Lipton, Allan	45	USA	Penn State Univ	6	Pienta, Kenneth J.	3,951	USA	Johns Hopkins Univ
7	Fizazi, Karim	44	France	Univ Paris Saclay	7	Brown, Janet E	3,690	England	Univ Sheffield
8	Tombal, Bertrand	43	Belgium	Clin Univ St Luc	8	Logothetis, Christopher J.	3,357	USA	Univ Texas MD Anderson Canc Ctr
9	Smith, Matthew R.	43	USA	Massachusetts Gen Hosp	9	Shore, Neal D	3,260	USA	Carolina Urol Res Ctr
10	Sartor, Oliver	42	USA	Tulane Canc Ctr	10	Guise, Theresa A	2,997	USA	Indiana Univ Sch Med

Lotka’s Law is considered as one of the most noted laws in bibliometric studies (23). It demonstrates the scientific productivity and the relation between the authors and the number of their outputs by predicting the contribution of an author for a publication. **Supplementary Figure 2A** confirms the expected pattern of scientific productivity, with 79.1% of authors contributing just one publication and fewer than 0.1% qualifying as core authors (≥25 publications). More details are shown in **Supplementary Table 2**.

Temporal analysis of author productivity (**Supplementary Figure 2B**) shows Saad Fred’s consistent annual output from 2004 to 2024, with peak productivity in 2018 (12 publications).

Co-citation analysis examines how frequently authors are cited together in subsequent research. This metric reveals both conceptual connections between researchers’ work and their collective influence in the field. Higher co-citation frequency indicates stronger thematic alignment and greater scholarly impact (**Figure 3C**). The authors were grouped into five distinct clusters: Saad Fred, Coleman RE, Smith Matthew R, etc. (green); Fizazi Karim, Parker C, etc. (blue); Roodman GD, Mundy GR, Lande R, etc. (red); Heidenreich A, Jemal A, etc. (yellow).

Further analysis of collaboration patterns (**Figure 3D**) reveals the interconnected nature of PCBM research teams. The center of the collaborative network is dominated by Saad Fred and Coleman RE. Saad Fred is actively associated with Christopher J. Logothetis, Toni Ibrahim, and Theresa A, while Coleman RE is closely collaborating with Karim Fizazi, Edward Chow, and Takeo Kosaka.

Publication type analysis (**Supplementary Figure 3A**) shows a significant predominance of research articles over review papers (P < 0.001). It reveals that articles constitute a substantial portion of each author’s publications.

### 3.4 Distributions of journals

**Figure 4:**
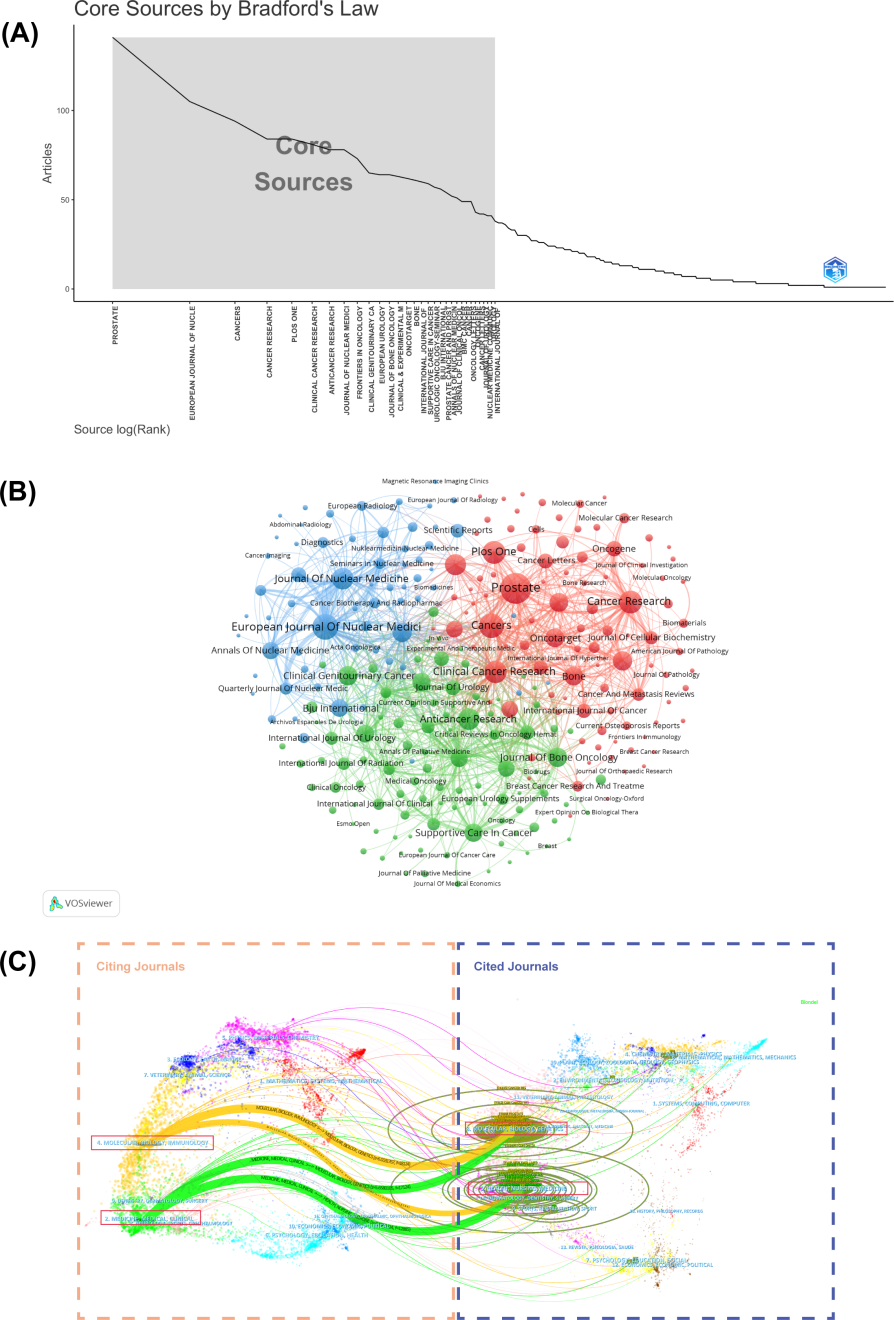
**(A)** Graphical representation of Bradford’s Law. **(B)** Cluster visual analysis of the journals’ cooperation network. Different nodes represent different journals; the lines between nodes indicate a cooperative relationship. **(C)** The dual-map overlay of citing journals on the left side and cited journals on the right. The vertical axis of the ellipse represents the journals’ output, and the horizontal axis of the ellipse represents the number of authors included.

The analysis of journal distribution reveals several important patterns in PCBM research dissemination. A total of 1,035 journals contributed to the publication of 5,995 articles, with Prostate (141 publications), European Journal of Nuclear Medicine and Molecular Imaging (105), and Cancers (94) emerging as the top three most prolific journals (**Table 4**). These core journals, along with 28 others, constitute the most productive sources in Zone 1 of Bradford’s Law distribution (**Figure 4A**, **Supplementary Table 3**).

**Table 4:** Top 10 journals related to prostate cancer bone metastasis research.

Rank	Journal	Publication	Rank	Journal	Total link strength	Rank	Journal	Citation
1	Prostate	141	1	Journal of Clinical Oncology	1,721	1	Journal of Clinical Oncology	9,838
2	European Journal of Nuclear Medicine and Molecular Imaging	105	2	Clinical Cancer Research	1,719	2	Clinical Cancer Research	8,450
3	Cancers	94	3	European Journal of Nuclear Medicine and Molecular Imaging	1,685	3	Cancer Research	6,907
4	Cancer Research	84	4	European Urology	1,476	4	European Urology	6,568
5	Plos One	84	5	Cancers	1,270	5	European Journal of Nuclear Medicine and Molecular Imaging	5,736
6	Clinical Cancer Research	81	6	Journal of Nuclear Medicine	1,223	6	Cancer	5,074
7	Journal of Nuclear Medicine	78	7	Prostate	1,206	7	Lancet Oncology	4,815
8	Anticancer Research	78	8	Cancer Research	1,201	8	Prostate	4,555
9	Frontiers in Oncology	73	9	Lancet Oncology	1,132	9	Journal of Nuclear Medicine	4,107
10	Clinical Genitourinary Cancer	65	10	Cancer	1,070	10	Plos One	2,923

Cluster analysis of journals with five or more publications identified three distinct groups (**Figure 4B**), reflecting different thematic orientations within the field. More importantly, a dual-map overlay of journals helps illustrate the dynamic and complex evolution of research fields, with journals categorized based on their publication themes (**Figure 4C**). There are four primary citation paths connecting the following domains: *Molecular/Biology/Genetics*, *Health/Nursing/Medicine*, *Molecular/Biology/Immunology*, and *Medicine/Medical/Clinical*. The intersection of citation paths between *Molecular/Biology/Genetics* and *Health/Nursing/Medicine* have given rise to two new research foci: *Molecular/Biology/Immunology*, and *Medicine/Medical/Clinical.* These cross-disciplinary patterns indicate that the PCBM research field is evolving toward a more integrated and multidisciplinary approach. This trend aims to deepen the understandings of the molecular mechanisms underlying health and disease, while exploring new avenues for targeted therapies and personalized medicine.

### 3.5 Distributions of reference

Reference analysis provides crucial insights into the intellectual foundations of PCBM research. The most influential work is the landmark study “Alpha Emitter Radium-223 and Survival in Metastatic Prostate Cancer”, published in the *New England Journal of Medicine*, with 2,377 citations and 216.09 citations per year (**Supplementary Table 4**, **Table 5**). This paper presents a pivotal phase III trial demonstrating that radium-223 improves overall survival in men with castration-resistant prostate cancer (CRPC) and BM compared to placebo (24).

**Table 5:** Top 15 annual citation publication in the field of prostate cancer bone metastasis.

Rank	First author	Year	Source	Article title	DOI	Citation	Document type	Citation per year
1	Parker C	2013	New England Journal of Medicine	Alpha Emitter Radium-223 and Survival in Metastatic Prostate Cancer	https://doi.org/10.1056/NEJMoa1213755	2,377	Article	216.09
2	Smith Matthew R	2018	New England Journal of Medicine	Apalutamide Treatment and Metastasis-free Survival in Prostate Cancer	https://doi.org/10.1056/NEJMoa1715546	883	Article	147.17
3	Kwon ED	2014	Lancet Oncology	Ipilimumab versus placebo after radiotherapy in patients with metastatic castration-resistant prostate cancer that had progressed after docetaxel chemotherapy (CA184-043): a multicentre, randomized, double-blind, phase 3 trial	https://doi.org/10.1016/S1470-2045(14)70189-5	1,157	Article	115.70
4	Heidenreich A	2014	European Urology	EAU guidelines on prostate cancer. Part II: Treatment of advanced, relapsing, and castration-resistant prostate cancer	https://doi.org/10.1016/j.eururo.2013.11.002	1,097	Review	109.70
5	Fizazi Karim	2011	Lancet	Denosumab versus zoledronic acid for treatment of bone metastases in men with castration-resistant prostate cancer: a randomized, double-blind study	https://doi.org/10.1016/S0140-6736(10)62344-6	1,410	Article	108.46
6	Coleman RE	2006	Clinical Cancer Research	Clinical Features of Metastatic Bone Disease and Risk of Skeletal Morbidity	https://doi.org/10.1158/1078-0432.CCR-06-0931	1,681	Review	93.39
7	Teo MY	2019	Annual Review of Medicine	Treatment of Advanced Prostate Cancer	https://doi.org/10.1146/annurev-med-051517-011947	423	Review	84.60
8	Roodman GD	2004	New England Journal of Medicine	Mechanisms of Bone Metastasis	https://doi.org/10.1056/NEJMra030831	1,688	Review	84.40
9	Stopeck AT	2010	Journal of Clinical Oncology	Denosumab Compared With Zoledronic Acid for the Treatment of Bone Metastases in Patients With Advanced Breast Cancer: A Randomized, Double-Blind Study	https://doi.org/10.1200/JCO.2010.29.7101	1,094	Article	78.14
10	Weilbaecher KN	2011	Nature Reviews Cancer	Cancer to bone: a fatal attraction	https://doi.org/10.1038/nrc3055	921	Review	70.85
11	Coleman RE	2020	Nature Reviews Disease Primers	Bone metastases	https://doi.org/10.1038/s41572-020-00216-3	267	Article	66.75
12	Gillessena S	2020	European Urology	Management of Patients with Advanced Prostate Cancer: Report of the Advanced Prostate Cancer Consensus Conference 2019	https://doi.org/10.1016/j.eururo.2020.01.012	267	Article	66.75
13	Nuhn P	2019	European Urology	Update on Systemic Prostate Cancer Therapies: Management of Metastatic Castration-resistant Prostate Cancer in the Era of Precision Oncology	https://doi.org/10.1016/j.eururo.2018.03.028	320	Review	64.00
14	Henry DH	2011	Journal of Clinical Oncology	Randomized, Double-Blind Study of Denosumab Versus Zoledronic Acid in the Treatment of Bone Metastases in Patients With Advanced Cancer (Excluding Breast and Prostate Cancer) or Multiple Myeloma	https://doi.org/10.1200/JCO.2010.31.3304	828	Article	63.69
15	Clézardin P	2021	Physiological Reviews	Bone metastasis: mechanisms, therapies, and biomarkers	https://doi.org/10.1152/physrev.00012.2019	162	Review	54.00

**Figure 5:**
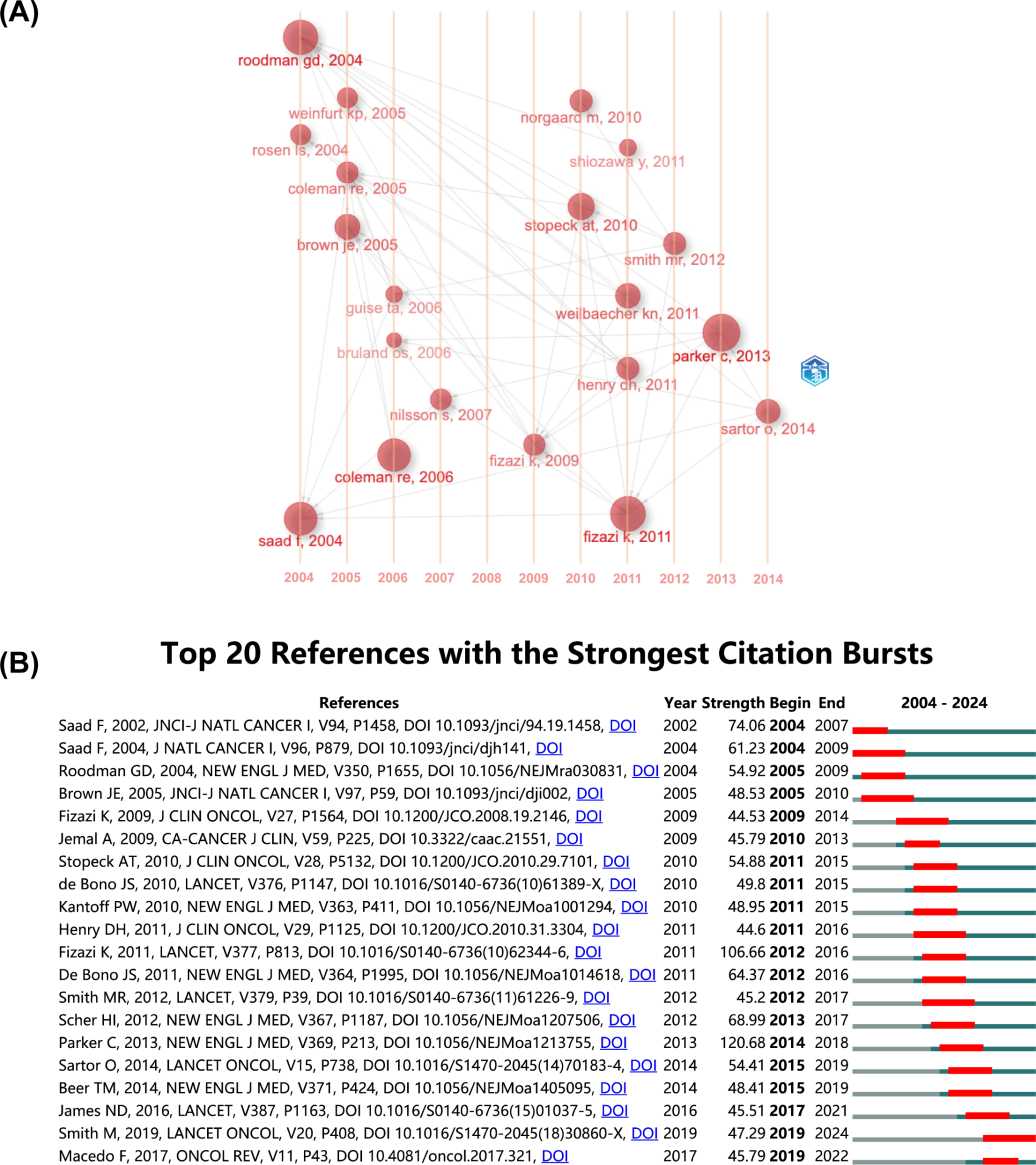
**(A)** The historiograph of the co-citation network. **(B)** Top 20 references with the strongest citation bursts. The blue line indicates the time interval, while the red line suggests the time period when a reference burst occurs.

The co-citation network historiograph (**Figure 5A**) identifies 20 seminal papers that have shaped the field’s development. Notably, citation patterns reveal that reviews are significantly more cited than original articles (P<0.001, **Supplementary Figure 3B**), suggesting that reviews are more likely to be used as reference materials in PCBM research.

A citation burst refers to a reference that undergoes a statistically significant surge in citations over a defined period. The top 20 with the strongest citation bursts were listed in **Figure 5B**. Generally, the most cited references also tend to experience the highest citation bursts. The strongest burst (strength=120.68) occurred in a paper entitled “Alpha Emitter Radium-223 and Survival in Metastatic Prostate Cancer”, published in journals by Christopher Parker, et al. in 2013 (24), with citation burst from 2014 to 2018. “Denosumab versus zoledronic acid for treatment of bone metastases in men with castration-resistant prostate cancer: a randomized, double-blind study” by Karim Fizazi, et al. published in journals in 2011, also had a high burst (Strength=106.66). According to the findings, a surge appears in citations 1–2 years post-publication, with two initial bursts occurring in 2004, one still active, and the majority peaking between 2012 and 2016.

### 3.6 Analysis of keywords

**Figure 6:**
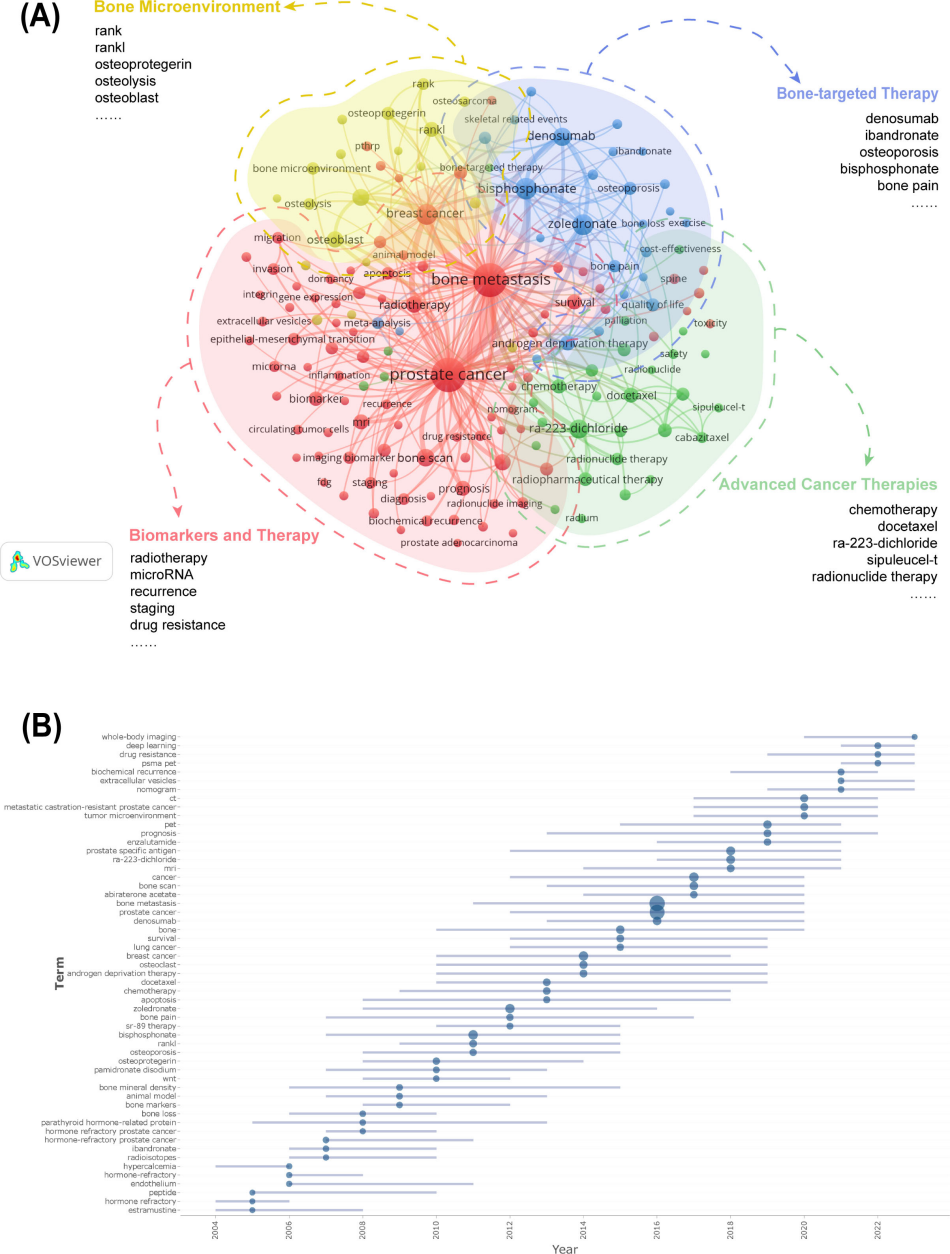
**(A)** Cluster analysis of keyword co-occurrence. **(B)** Cluster analysis of keywords. Keywords from the authors were analyzed, examining three per author annually, each appearing at least five times. The blue line suggests the duration of occurrences for each topic, while the circle indicates the frequency of each topic.

Keyword analysis reveals both established and emerging research directions in PCBM. The 20 most frequent keywords were sorted by occurrence, just as **Table 6** shows. The keywords were clustered into four thematic groups (**Figure 6A**): The red for biomarkers and therapy with terms like “radiotherapy” and “microRNA”; the green for advanced cancer therapies with “chemotherapy” and “docetaxel”; the blue for bone-targeted therapy with “denosumab” and “ibandronate”; and the yellow for bone microenvironment with “zoledronate” and “bone pain”. The overlay of the historical trend for keywords (**Supplementary Figure 4**) highlights “ra-223-dichloride”, “biomarker” and “biochemical recurrence” as the frequent keywords in the past 5 years, suggesting that they may be the focus of future research.

**Table 6:** Top 20 keywords in the research of prostate cancer bone metastasis.

Rank	Keyword	Total link strength	Occurence	Rank	Keyword	Total link strength	Occurence
1	prostate cancer	3,520	2,119	11	prostate specific antigen	281	138
2	bone metastasis	3,712	1,983	12	osteoblast	315	125
3	bisphosphonate	904	356	13	mri	179	104
4	breast cancer	874	341	14	androgen deprivation therapy	260	103
5	zoledronate	865	319	15	survival	223	99
6	ra-223-dichloride	454	219	16	prognosis	189	97
7	denosumab	656	213	17	docetaxel	231	94
8	bone scan	354	182	18	rankl	318	94
9	osteoclast	402	146	19	chemotherapy	209	88
10	radiotherapy	260	139	20	biomarker	167	82

Trend topic analysis serves as a powerful bibliometric tool for mapping conceptual evolution within research domains by clustering semantically related keywords into thematic constructs. The trend analysis of author keywords in PCBM research (**Figure 6B**) demonstrates a dynamic research landscape where conventional clinical approaches coexist with cutting-edge innovations. The most frequently occurring terms (>100 mentions) over the past five years predominantly represent established diagnostic modalities (“prostate specific antigen”, “bone scan”, “PET”, “CT”, “MRI”), therapeutic agents (“Ra-223-dichloride”, “denosumab”), and disease states (“metastatic castration-resistant prostate cancer”), reflecting current mainstream practices. Simultaneously, we observe the rapid emergence of transformative research directions including advanced imaging techniques (“PSMA PET,” “whole-body imaging”), artificial intelligence applications (“deep learning”), molecular investigations (“extracellular vesicles,” “drug resistance”), and clinical prediction tools (“nomogram”).

**Figure 7:**
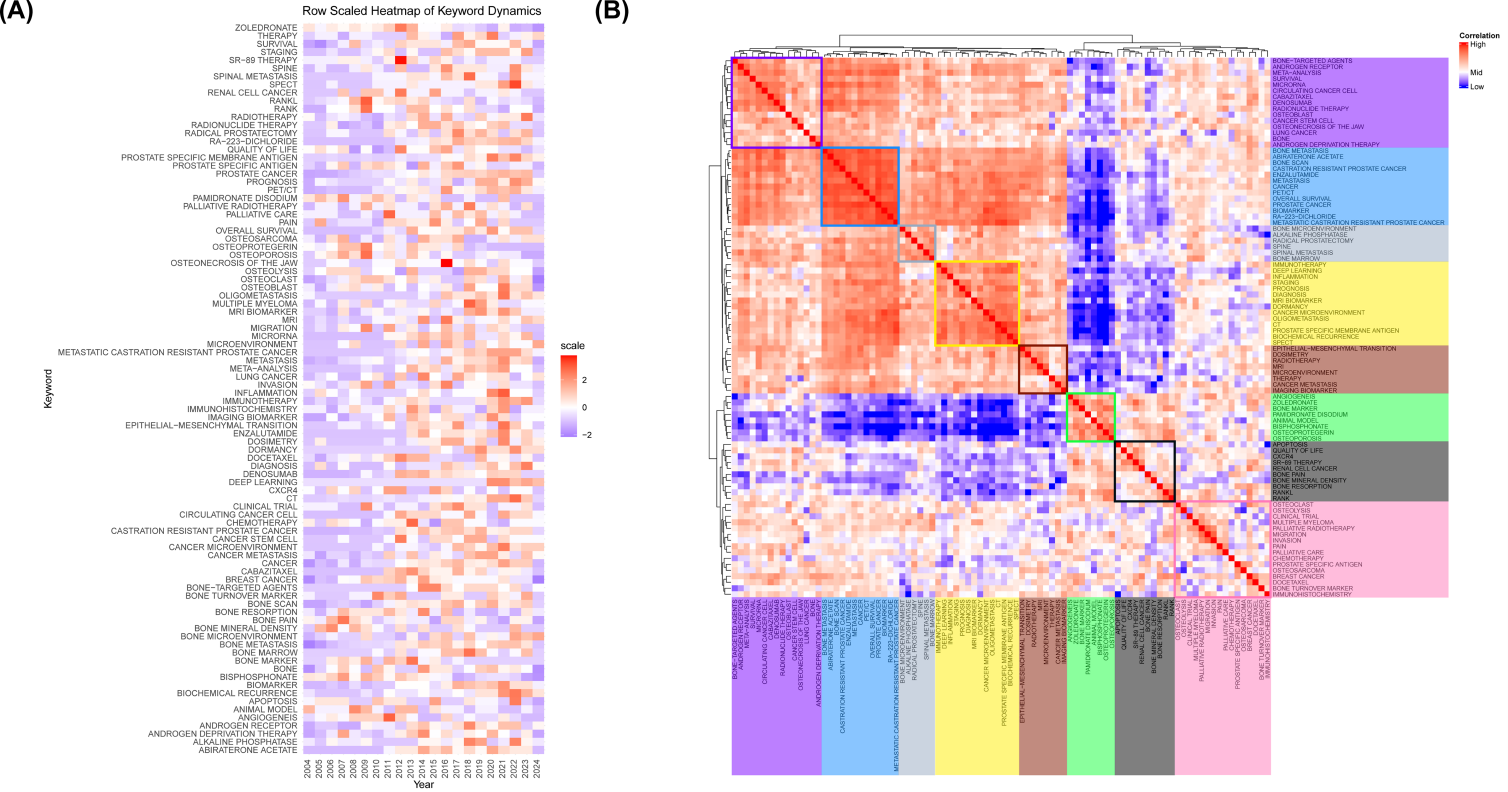
**(A)** Annual heatmap from 2004 to 2024. Each keyword’s prominence for a given year is determined by the number of times it is cited relative to the total citations for that year. **(B)** Relevance heatmap of keywords. Keywords that are frequently cited concurrently are clustered and color-coded to represent their thematic associations.

**Figure 7A** displays the annual usage patterns of keywords in PCBM research from 2004 to 2024. This metric was calculated as the ratio of keyword-specific citations to total citations per year. A marked increase has been observed in the popularity of keywords including “biochemical recurrence”, “immunotherapy”, “metastatic castration-resistant prostate cancer”, and “deep learning” over the past five years, confirming the evolving research trends shown in **Figure 6B**. In contrast, keywords such as “bone marker” and “osteoporosis” have maintained relatively low annual popularity.

A popularity correlation analysis represents distinct but interconnected research fronts. In **Figure 7B**, keywords with high popularity in the same period are clustered together in a category and denoted by distinct colors. There are 8 clusters: the rosy cluster (“bone-targeted agents”, “androgen receptor”, “meta-analysis”, etc.), the blue cluster (“bone metastasis”, “abiraterone acetate”, “bone scan”, etc.), the grey cluster (“bone microenvironment”, “alkaline phosphatase”, “radical prostatectomy”, etc.), the yellow cluster (“immunotherapy”, “deep learning”, “inflammation”, etc.), the brown cluster (“epithelial-mesenchymal transition”, “dosimetry”, “radiotherapy”, etc.), the green cluster (“angiogenesis”, “zoledronate”, “bone marker”, etc.), the black cluster (“apoptosis”, “quality of life”, “CXCR4”, etc.), and the pink cluster (“osteoclast”, “osteolysis”, “clinical trial”, etc.). It suggests that keywords within the same cluster represent significant areas of research focus during a specific period.

### 3.7 Analysis of GO and KEGG enrichment

**Figure 8:**
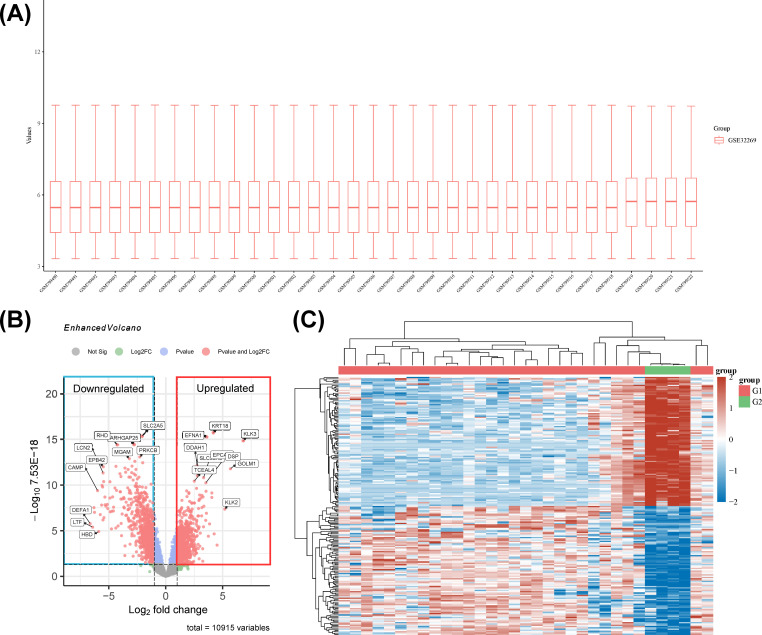
**(A)** The boxplot of the normalized data. Rows represent samples, and columns represent the gene expression values in the samples. **(B)** Volcano plot: The volcano plot was constructed using the fold change values and P-adjust. Dots on the left indicate downregulated genes; dots on the right indicate upregulated genes. **(C)** The heatmap of the differential gene expression, different colors represent the trend of gene expression in different tissues. The top 50 up-regulated genes and top 50 down-regulated genes were showed in this figure.

To dissect the molecular underpinnings of PCBM, we analyzed dataset GSE32269 from the GEO database, comparing 29 samples from patients with BM to 4 normal samples (**Figure 8A**). Applying a stringent cutoff with the limma package, we identified 936 upregulated and 1273 downregulated genes, marking a stark molecular divergence. The top 10 genes by expression level include TMOD1, KRT18, SLC2A5, EFNA1, KLK3, ARHGAP25, RHD, PRKCB, MGAM, and GFI1. Among these, TMOD1, SLC2A5, ARHGAP25, RHD, PRKCB, MGAM, and GFI1 are downregulated, while KRT18, EFNA1, and KLK3 are upregulated. These DEGs are visualized in a volcano plot and heatmap in **Figures 8B, C**, respectively. (Each detail of genes is attached in **Supplementary File 2**).

**Figure 9:**
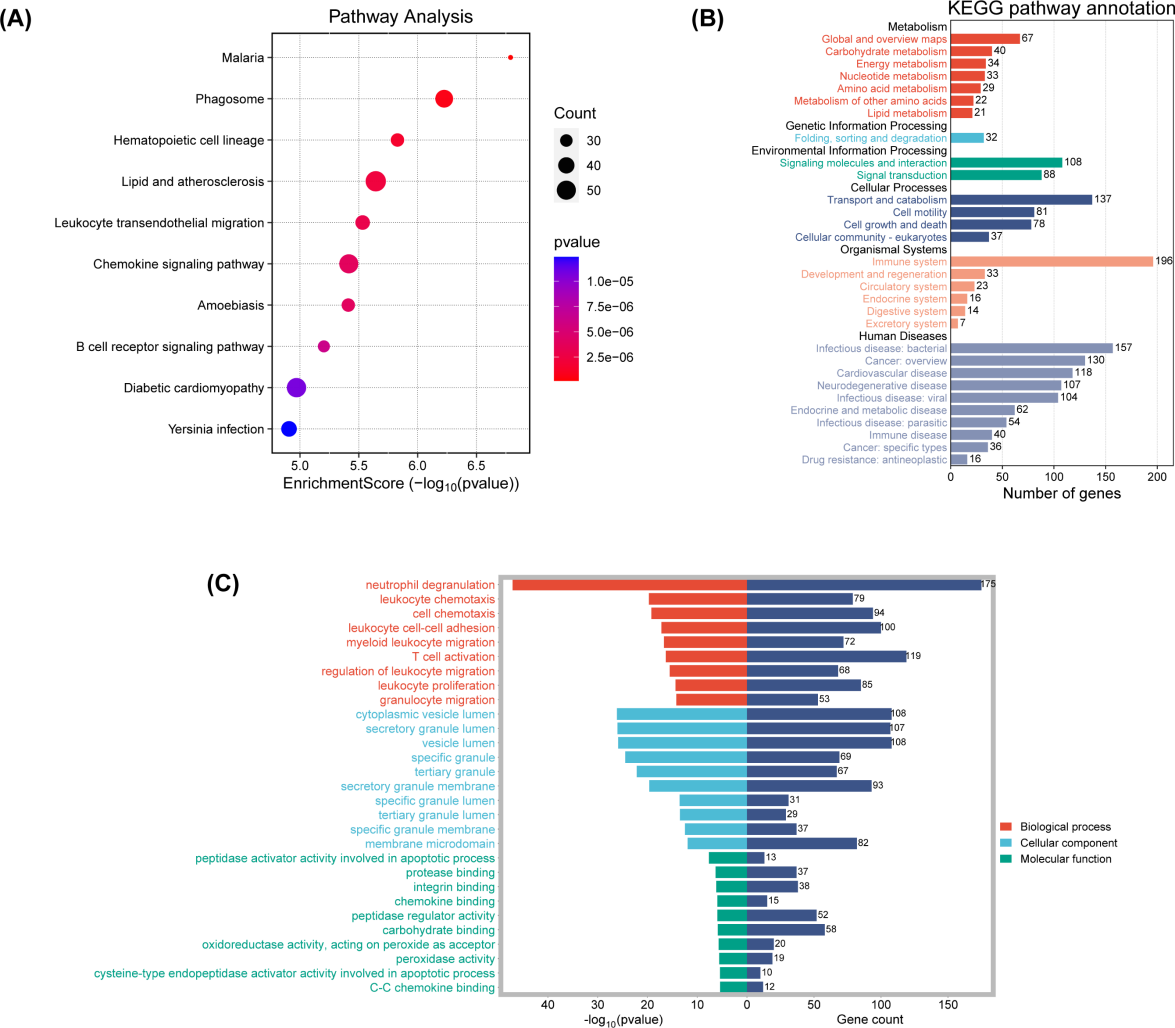
**(A)** The enriched KEGG signaling pathways were selected to demonstrate the primary biological actions of major potential mRNA. The abscissa indicates gene ratio and the enriched pathways were presented in the ordinate. **(B)** KEGG pathway annotation result. **(C)** GO enrichment for the cluster analysis of the pathways related to biological processes, cellular components and molecular functions. Horizontal axis denoted the respective -Log10 (p-value) of different pathways and the vertical axis represented the pathway name. Numbers of genes in each cluster are shown.

To unravel the biological narratives woven by these DEGs, GO and KEGG enrichment analyses were conducted. KEGG pathway analysis illuminated 94 significant pathways, with a curated subset of the top 10 pathways presented in a bubble plot (**Figure 9A**). Notably, pathways associated with malaria, phagosome processes, and hematopoietic cell lineage were significantly enriched, highlighting the intricate interplay between tumorigenesis and immune surveillance.

A gene-concept network (**Supplementary Figure 5A**) revealed significant interconnections between key genes such as IL1B and IL10 and multiple pathways, suggesting a pivotal role for interleukins in diverse biological processes. The chemokine family (CCR2, CCL18, etc.) and the leukocyte immunoglobulin-like receptor (LILR) family (LILRB1, LILRA2, etc.) were also significantly enriched.

KEGG pathway enrichment analysis, stratified and visualized, facilitated the identification of enriched pathways (**Figure 9B**). Genes under the “Organismal Systems” category were significantly enriched, potentially linking to the tumor’s immune evasion strategies. Upregulation in the “Human Diseases” category, particularly in cardiovascular and neurodegenerative diseases, suggests shared pathways or therapeutic targets in PCBM. The “Genetic Information Processing” and “Environmental Information Processing” categories also showed significant upregulation, indicating a nexus with genetic expression and signaling activation in metastatic progression.

GO functional enrichment analysis was then conducted, with the results shown in **Figure 9C** and **Supplementary Figure 5B**. The bar charts highlight the top 10 most significantly different components in each of the three functional categories. In the “Biological Process” category, neutrophil degranulation, leukocyte chemotaxis, cell chemotaxis, T cell activation, and leukocyte proliferation are significantly enriched. The cnetplot of biological functions (**Supplementary Figure 5B**) reveals the complex relationships between related genes and biological functions.

**Supplementary File 3** contains a comprehensive category information.

### 3.8 Analysis of related genes and diseases

The BioBERT biomedical domain-specific language representation model was used to mine and statistically analyze the genes and diseases from entity words of genes and diseases in the abstracts of PCBM articles. The top five most documented genes were KLK3, TNFSF11, NPEPPS, AR, TGFB1 (**Supplementary Figure 5C**). Moreover, the top five diseases by frequency of occurrence were prostate cancer (including mCRPC and CRPC), neoplasms, neoplasm metastasis, bone neoplasms, and bone diseases (**Supplementary Figure 5D**).

## 4 Discussion

### 4.1 General distribution

#### 4.1.1 Global research landscape

The annual publications from 2004 to 2024 have generally shown an upward trend, suggesting that the research of PCBM has still a lot of potential research directions. In **Table 1** and **Supplementary Figure 1A**, we saw that the USA distributes the most articles in this field, as well as the strongest link strength and the most citations, affirming its dominance in this field. While China ranks second in publications, reflecting its recent commitment to this domain (**Supplementary Figure 1C**), England and Germany have achieved higher citations and stronger link strengths, being early contributors to the field. This suggests that Chinese research on PCBM still needs broader recognition and evaluation globally and will require sustained effort and time. The fact that publication volume varying from countries/regions is may be resulting from the differences in PC incidence in different countries/regions. The observed geographic disparities in prostate cancer bone metastasis (PCBM) research output appear to parallel regional variations in disease burden, though with notable exceptions. Analysis of GLOBOCAN 2022 data (1, 25), reveals that crude rates in America (138.92 per 100,000), the United Kingdom (163.79), and Germany (157.23) generally align with concentrated research productivity in these regions, as demonstrated by the synergy between **Figure 2B** and **Table 1**. While high-income nations like the United States and United Kingdom exhibit both substantial disease prevalence and dominant publication outputs, this correlation breaks down in Latin America and the Caribbean – regions with comparable incidence rates (e.g., 188.38 per 100,000 in Barbados) but disproportionately low research contributions (<3% of total publications). More detailed information is attached in the **Supplementary File 1**. This discrepancy likely stems from socioeconomic disparities, where limited healthcare funding and competing public health priorities constrain research infrastructure development. Compounding this challenge, delayed implementation of population-wide PSA screening programs and fragmented cancer registry systems in these regions may obscure the true PCBM burden, perpetuating a cycle of underinvestment. These findings highlight the critical need for global oncology initiatives to prioritize capacity-building partnerships that address both biological and systemic determinants of research inequity. The predominance of Western populations in datasets like GSE32269 raises questions about the generalizability of molecular mechanisms across ethnicities. Future studies should prioritize diverse cohorts to uncover population-specific drivers of PCBM. Geographic concentration of research output (**Figure 3A**) also reflects systemic inequities. Funding disparities and language barriers—evidenced by the underrepresentation of non-English studies in WoSCC—limit global collaboration. Initiatives like open-data repositories and multilingual publishing could democratize participation.

In terms of institutions, as shown in **Table 2** and **Supplementary Figure 1D**, approximately 70% of the top 10 affiliations are based in the United States, with the remaining being in England (n=2) and Canada (n=1). The distribution of institutions confirms America being a central leader in this field. Additionally, though China has the second most publications, the institutions in China are not in the rank of top 10 affiliations, and the most productive institution is in rank 40.

Based on the above analysis, PCBM research in China appears to be relatively dispersed and has not yet reached a systematic or large scale. Therefore, institutions and universities in China should concentrate more resources to support related research.

However, despite **Figure 3A** showing relatively close cooperation between institutions, research remains highly geographically concentrated, and active participation in international collaborative exchanges is still limited. We advocate for fostering collaboration in this domain that surpasses geographical and political boundaries and encourage researchers to proactively pursue further cooperative interactions with their global peers.

In recent years, against the backdrop of an aging population and rising incidence of PC, it is imperative for researchers and clinicians to urgently improve the prognosis of mPC.

Among the top 10 most productive journals, *Prostate* has published the most research on PCBM (**Figure 4B**, **Table 4**). Besides *Prostate*, *European Journal of Nuclear Medicine and Molecular Imaging*, *Journal of Nuclear Medicine*, and *Clinical Cancer Research* are the top 10 most published and co-cited journals with strongest link strength. These academic journals have played a crucial part in this domain as core journals evaluated by Bradford’s Law (**Figure 4A**).

The 2010–2016 period saw particularly rapid growth in publications (**Figure 5B**), coinciding with two landmark therapeutic advances: 1) the 2010 FDA approval of denosumab for preventing skeletal-related events, supported by Phase III trial data (NCT00321620) showing significant reductions in vertebral fractures (26); and 2) the 2013 ALSYMPCA trial demonstrating survival benefits of radium-223 in metastatic castration-resistant prostate cancer (24). These breakthroughs not only drove citation bursts but also established bone-targeted therapies as a research priority.

#### 4.1.2 Research keyword insights

During the first 15 years of the past 20 years, major trends in PCBM research have focused on understanding the mechanisms of PCBM and managing bone-related complications. Research shows hotspots include bone pain, bone density, bone resorption, osteoporosis, and osteoprotegerin, which are designed to alleviate bone pain and prevent bone loss in patients.

In the **Figures 7A, B**, we found that around 2010, the four keywords “RANK”, “RANKL”, “osteoprotegerin”, “osteoporosis” had a significant increase in the research heat in this field, which was due to the fact that back in 2009 Smith, et al. reported for the first time on a randomized controlled trial of de-escalation-treated patients with non-metastatic prostate cancer (nmPC) using a targeted RANK/RANKL pathway after denosumab treatment increases bone mineral density and reduces the risk of new vertebral fractures (27). Subsequently, in a 2011 randomized, double-blind trial on denosumab versus zoledronic acid control, Henry, et al. reported that in patients with BM from advanced cancers (breast, prostate) and multiple myeloma, there was a significant difference in reducing or preventing skeletal-related events (28). Based on the above and related clinical findings, the FDA (U.S. Food and Drug Administration) approved denosumab for osteoporosis (postmenopausal osteoporosis) in 2010 and the EMA (European Medicines Agency) approved denosumab for delaying the onset of bone-related events in patients with metastatic cancer, and for bone loss induced by aromatase and androgen deficiencies in 2011. In the same year, research related to animal models in the field of PCBM was hot, and the study by Jimenez, et al. introduced a variety of novel models of bone metastatic cancer pain, which provided fresh insights into the mechanisms of pain generation in patients with BM and will provide new ideas for therapies based on mechanism-based studies (29).

As the research progressed, in a meta-analysis covering three large randomized controlled trials (RCTs) published in 2012, denosumab was superior to zoledronic acid in preventing the development of skeletal related events (SREs) in patients with BM from advanced cancers, with a favorable safety profile, which explains the outbreak of studies that year that used zoledronic acid as the keyword (30).

Before and after 2010, it was an era of blossoming in the field of PCBM research, with new advances in treatments in the field of conventional radiotherapy (31), as well as continued new explorations of the pathogenesis (32) and therapeutic targets of PCBM (33). These fruitful research results, besides laying a theoretical foundation for future research, have greatly enriched the treatment of PCBM, provided more treatment options for patients, and also brought new hope and direction to the field of PC treatment. The explosive emergence of “PSMA PET” (**Figure 6B**) directly correlates with its 2020 FDA approval for metastatic lesion detection, as this technology enables precise localization of bone metastases to guide radiotherapy. The rise of “deep learning” reflects breakthroughs in AI applications for pathological slide analysis (e.g., P-NET models) and radiomics.

In recent years, research has shifted to a more precise and molecular level of exploration. mCRPC has become a focal point of research, with researchers working to understand and overcome mechanisms of treatment resistance. Based on the trend topic analysis presented in **Figure 6B**, six research keywords are currently gaining significant momentum: “whole-body imaging,” “deep learning,” “drug resistance,” “PSMA PET,” “nomogram,” and “extracellular vesicles.” Their collaborative evolution is reshaping the research paradigm of prostate cancer bone metastasis (PCBM). The integration of PSMA PET with whole-body imaging represents a dual breakthrough: clinically, it achieves higher diagnostic sensitivity, enabling simultaneous detection of lymph node, bone, and visceral metastases in intermediate- and high-risk prostate cancer; in translational medicine, the theranostic properties of PSMA-targeted ligands are driving the iterative development of novel radioligand drugs. Deep learning with artificial intelligence enhances the specificity of early bone metastasis detection through the deep integration of radiomic feature extraction and transfer learning frameworks. Notably, extracellular vesicles, acting as “molecular postmen” in the tumor microenvironment, can advance the warning window for biochemical recurrence when combined with other biomarkers. Additionally, by modulating multiple downstream pathways, they offer a new perspective for overcoming current therapeutic bottlenecks. This triangular synergy of technology, mechanism, and tools signifies that PCBM research has entered a phase of systematic innovation.

The technological renaissance in prostate cancer management—spearheaded by liquid biopsy-enabled circulating tumor DNA dynamics tracking, next-generation sequencing (NGS)-guided clonal architecture mapping, and spatially resolved high-throughput histopathology—has fundamentally redefined diagnostic paradigms. This multidimensional profiling capacity has catalyzed two paradigm shifts: Firstly, the emergence of bone marrow immune ecology as a therapeutic frontier, where single-cell RNA sequencing uncovers them crosstalk between dormant tumor cells and osteal macrophages; and secondly, precision immunotherapy strategies leveraging AI-optimized neoantigen prediction from PSMA PET radiomic features. Notably, it is predicted that growing recognition of microenvironmental reprogramming will be the next therapeutic axis. These converging technological and conceptual advances position PCBM research at the threshold of mechanism-guided therapeutic innovation.

#### 4.1.3 Key genes and pathways

GO and KEGG enrichment analyses indicate that these DEGs may play diverse roles in the process of BM. Changes in the tumor immune microenvironment, such as the secretion of cytokines and chemokines by tumor-associated immune cells, the binding of these factors to their receptors, and alterations in extracellular matrix components, are crucial. The leukocyte transendothelial migration pathway’s enrichment suggests a role in tumor immune evasion and immune cell trafficking. Chemokine signaling pathway activation likely orchestrates immune cell recruitment, while B cell receptor signaling pathway enrichment may shape humoral immune responses. Pathways related to diabetic cardiomyopathy and Yersinia infection may reflect metabolic reprogramming and inflammatory responses critical to metastatic progression.

The enrichment of the leukocyte immunoglobulin-like receptors (LILRs) family is critical for regulating various components of the tumor immune microenvironment (34). LILRs may be involved in immune checkpoint inhibition processes and the production of tumor-sustaining factors. PC is often considered an “immune desert” due to its unique tumor microenvironment, which makes chemotherapy and immunotherapy less effective. Simultaneously, as it shown in **Supplementary Figure 5B**, for example, leukocyte chemotaxis is associated with multiple genes, indicating frequent leukocyte migration during BM. Additionally, most genes shown in the figure participate in multiple biological processes, demonstrating their multifunctionality in the BM process, such as CXCR2, which is involved in almost all functions. The findings of our study may explain some factors contributing to the “immune desert” phenomenon in mPC (35, 36).

Additionally, our results in **Supplementary Figure 5C** identified the top five most documented genes in PCBM as KLK3, NPEPPS, AR, TGFB1, PTHLH and AKT1. These genes are known to be involved in various aspects of tumor progression and metastasis. For instance, KLK3 (also known as PSA) is a well-established marker for PC (37), while TNFSF11 (RANKL) plays a critical role in bone remodeling and metastasis (38). The identification of these genes highlights their potential as therapeutic targets and biomarkers for PCBM. Moreover, the top five diseases associated with PCBM shown in **Supplementary Figure 5D** were identified as different kinds of diseases. This reinforces the complexity and multifaceted nature of PCBM, involving not only the primary tumor but also secondary complications and other related conditions. Understanding these associations can provide insights into the broader impact of PC on patient health and guide the development of comprehensive treatment strategies.

By integrating bibliometric and bioinformatics analyses, this study reveals profound connections between research trends and molecular mechanisms in prostate cancer bone metastasis (PCBM). High-frequency keywords identified through bibliometrics, such as ‘immunotherapy’ and ‘T cell activation,’ exhibited significant alignment with KEGG-enriched pathways, including the chemokine signaling pathway and B cell receptor signaling pathway. For instance, genes like KRT18 and KLK3 were markedly upregulated in differential expression analysis, while their roles in recruiting immunosuppressive cells (e.g., Tregs) were recurrently highlighted in keyword clusters such as ‘tumor microenvironment,’ suggesting their critical involvement in promoting bone metastasis. Furthermore, the explosive growth of emerging technical keywords like ‘PSMA PET’ (post-2018) corresponds to the enrichment of the phagosome pathway, as PSMA-targeted imaging relies on lysosome-mediated endocytosis—a key process annotated in this KEGG pathway. This bidirectional validation between technological advancements and molecular mechanisms underscores the unique value of interdisciplinary integration in deciphering the complex biology of PCBM.”

Our findings suggest that targeting these key genes and understanding their roles in the broader context of mPC could lead to more effective therapeutic strategies. By disrupting the pathways and interactions that facilitate BM, it may be possible to improve patient outcomes and reduce the burden of metastatic disease.

### 4.2 Hotspots and frontiers

Building upon the bibliometric trajectories identified in Section 4.1.2 – particularly the 2010 RANKL research surge (**Figure 7A**) and post-2020 molecular technology adoption (shown in **Figure 6B**) – current PCBM research converges on three transformative frontiers that bridge mechanistic discovery to clinical innovation. In recent years, research has increasingly focused on more precise and molecular-level explorations. mCRPC has become a focal point, with researchers striving to understand and overcome treatment resistance mechanisms.

The convergence of multimodal diagnostic technologies—spanning liquid biopsy-driven circulating tumor cell (CTC) enumeration, next-generation sequencing (NGS)-based genomic profiling, high-throughput proteomic/transcriptomic platforms, and multiplex immunohistochemistry (IHC)—has catalyzed a paradigm shift in prostate cancer bone metastasis (PCBM) management. These technologies enable researchers to better identify the genetic characteristics of individual patients, thereby formulating personalized treatment plans. This technological triad not only redefines precision oncology through patient-specific molecular cartography but also unveils previously inaccessible therapeutic vulnerabilities within the PCBM microenvironment, setting the stage for the following critical discussions on emerging frontiers.

#### 4.2.1 Multidisciplinary treatment strategies for PCBM: emerging biomarkers and imaging techniques for diagnosis, staging, and prognosis

Multidisciplinary treatment strategies for PCBM include emerging biomarkers and advanced imaging techniques. Biomarkers such as circulating tumor cells (CTCs), circulating tumor DNA (ctDNA), circulating tumor RNA (ctRNA), and extracellular vesicles (EVs) play crucial roles in the early diagnosis and prognosis of the disease (39). CTCs can be isolated from the patient’s blood using liquid biopsy techniques, providing real-time dynamic information about the tumor (40). EPISPOT is a method that detects viable CTCs based on the secretion, shedding, and release of specific tumor-associated proteins, and it has been validated in PC, demonstrating its clinical value (41). Circulating nucleic acids, including ctDNA and ctRNA, are free nucleic acid fragments released into the blood by cells, including tumor cells. Currently, the early diagnosis of PC primarily relies on the detection of prostate-specific antigen (PSA) in the blood. Long non-coding RNA (lncRNA PCA3) is a well-studied lncRNA that is highly expressed in PC tissues and can be detected in urine, already used for early diagnosis (42). A model constructed using six lncRNA detections has been confirmed to predict late biochemical recurrence of PC (43). After further clinical trials, it may be considered a potential clinical decision-making tool. Imaging techniques such as PET-CT and MRI provide high-resolution tumor localization and staging information. Using radiotracers based on prostate-specific membrane antigen to label tumor cells, PET-CT scans can provide comprehensive information on tumor distribution throughout the body. This precise identification of metastatic sites can guide lymph node dissection and subsequent radiotherapy (44). MRI, with its high-resolution soft tissue imaging, provides detailed tumor structure information. The combination of these technologies can significantly improve the diagnostic accuracy and treatment efficacy of PCBM (45). Additionally, new imaging techniques such as dual-energy X-ray absorptiometry (DXA) and ultrasound elastography (USE) show potential in the diagnosis of BM. DXA can assess the risk of osteoporosis by measuring bone density (46). USE can provide information on tumor stiffness by measuring tissue elasticity (47). Overall, multidisciplinary treatment strategies for PCBM offer new tools and methods for disease diagnosis, staging, and prognosis.

#### 4.2.2 Precision diagnosis and treatment of PCBM: the role of artificial intelligence and deep learning models in disease diagnosis and treatment

Our trend topic detection revealed a significant increase in “deep learning” related studies since 2020 (**Figure 6B**), mirroring the P-NET model’s breakthrough in overcoming traditional diagnostic bottlenecks. Artificial intelligence (AI) and deep learning models show great potential in the precision diagnosis and treatment of PCBM. AI algorithms can analyze large amounts of medical imaging data, providing accurate diagnostic and staging information. For example, deep learning-based MRI image analysis models can significantly improve the diagnostic accuracy of PC. These models, by learning from large amounts of annotated data, can automatically identify and classify tumor regions, reducing human error (48). Additionally, AI can be used to predict treatment responses and develop personalized treatment plans, thereby improving treatment outcomes. For instance, machine learning-based predictive models can analyze patients’ gene expression data to predict their response to specific drugs. This personalized treatment approach can significantly enhance treatment efficacy and reduce side effects (49). Recently, a predictive model named P-NET, which uses molecular data to predict PC status, has outperformed other modeling methods and can achieve preclinical detection and prediction of PC through neural network learning (50). Moreover, AI can monitor tumor changes during treatment, providing real-time treatment feedback. For example, deep learning-based imaging analysis models can automatically detect tumor changes before and after treatment, assessing treatment efficacy (51).

#### 4.2.3 Inflammatory molecular mechanisms, immunohistochemical analysis, and immunotherapy strategies of PCBM

Inflammation is a defense mechanism evolved in humans, serving as a barrier against pathogen invasion and a critical strategy for tissue repair and adaptation to environmental stress (52). It has been reported that the formation of tumor stroma shares similarities with the inflammation and tissue remodeling mechanisms observed in wound healing, which has led to the concept of tumors as “wounds that do not heal” (53). In recent years, the inflammatory molecular mechanisms of PCBM have garnered significant attention (54–56). The inflammatory microenvironment promotes tumor cell growth and metastasis through various pathways. Immunohistochemical analysis reveals significant immunosuppression at the sites of PCBM, primarily due to the secretion of various cytokines by tumor cells, such as TGF-β (57) and IL-6 (58). These cytokines not only inhibit T cell activity but also promote the accumulation of immunosuppressive cells such as regulatory T cells (Tregs) and myeloid-derived suppressor cells (MDSCs) (59, 60). Additionally, studies have shown that PC cells can secrete chemokines such as CCL2 (61, 62) and CXCL12 (63, 64), attracting immunosuppressive cells to the BM sites, thereby enhancing the immunosuppressive effect (65). Targeting IL-6 (e.g., siltuximab) or TGF-β (e.g., galunisertib) could disrupt the PCBM microenvironment, while PD-L1 inhibitors (e.g., pembrolizumab) may enhance radioligand efficacy. Clinical trials exploring these combinations (NCT04071236, NCT03873805) are underway.

The process of cancer bone metastasis is often accompanied by systemic or local inflammatory responses, as well as a locally immunosuppressive environment at the metastatic sites. These factors collectively form a positive feedback loop between inflammation and epithelial-mesenchymal transition (EMT) (58, 66). Inflammatory mediators, such as cytokines IL-1β (67, 68), IL-6 (69), IL-8 (70, 71), and chemokines CCL2 (72), CCL5 (73), promote EMT progression, driving tumor cell metastasis and colonization. Specifically, the pro-inflammatory cytokine IL-6 can activate pathways such as STAT3 (74), PI3K (75), and MAPK (75), which play crucial roles in bone remodeling, inflammatory responses, and tumor cell survival and proliferation. Furthermore, IL-6 can upregulate the expression of cyclooxygenase (EP) receptors and mediate the inhibition of osteoprotegerin secretion induced by prostaglandin E2, further affecting bone metabolism (76). IL-6, in combination with its soluble receptor sIL-6R, can both promote and inhibit osteoclast differentiation, thus playing a dual role in BM (76).

Overall, interleukins play a key role in controlling bone remodeling during normal bone metabolism and the BM process, making them potential targets for future PCBM treatments. Humanized monoclonal antibodies targeting soluble and membrane-bound IL-6 receptors, such as cetuximab, have shown efficacy in inhibiting PC cell proliferation, reducing tumor burden in animal models, and improving survival rates (77). Additionally, cetuximab effectively inhibits the transition of androgen-dependent PC to more aggressive mPC (78), and reduces inflammatory markers in the blood, such as CRP, indicating decreased inflammation and positive therapeutic outcomes (75, 79, 80).

In terms of immunotherapy strategies, combination therapies, vaccine treatments, and novel drug research offer new hope for treating PCBM. For instance, using PD-1/PD-L1 inhibitors can restore T cell activity, thereby enhancing anti-tumor immune responses (81). CTLA-4 inhibitors have also shown efficacy by blocking the CTLA-4 signaling pathway, reducing the number of Treg cells, and enhancing effector T cell function (82). A phase II clinical study combining CTLA-4 and PD-1 dual inhibition in treating mCRPC demonstrated superior efficacy compared to single immune checkpoint inhibitors (83). Sipuleucel-T, an autologous cellular immunotherapy, stimulates the body to produce tumor antigen-specific anti-tumor immune responses and is clinically used to treat mCRPC (84, 85). Recent advancements show that PSCA-CAR T cell therapy exhibits initial safety and bioactivity in treating mCRPC. The high expression of prostate stem cell antigen(PSCA) in PC, especially in BM, makes it an attractive therapeutic target (86). Phase I clinical trial results support future clinical studies to optimize dosage and combination strategies, further improving the durable treatment outcomes for mCRPC patients (87).

Since Hanahan et al. proposed tumor-promoting inflammation as an emerging hallmark of cancer in 2011, research on the inflammatory molecular mechanisms and tumor immune microenvironment has become a mainstream direction in PCBM studies (54). However, research on interleukin-targeted therapies is still in its early stages, requiring further elucidation of the roles of various inflammatory mediators in BM and related downstream pathways. The inflammatory molecular mechanisms and immunohistochemical analysis of PCBM provide important theoretical foundations and practical guidance for immunotherapy strategies. The emergence of various novel immunotherapy strategies offers new hope for treating PCBM, but their long-term clinical value remains under exploration, necessitating more data to validate the durability and long-term safety of these treatments.

#### 4.2.4 The tumor microenvironment of PCBM: disruption of the balance between osteoblasts and osteoclasts

Emerging from the RANKL/zoledronic acid debate, current research transcends simple osteoclast inhibition to target microenvironmental cross-talk. The tumor microenvironment of PCBM is a complex ecosystem involving dynamic interactions among various cell types, including osteocytes, osteoclasts, osteoblasts, vascular endothelial cells, hematopoietic stem cells, mesenchymal stem cells, and growth factors (88, 89). These interactions significantly influence tumor cell colonization and activation, with the imbalance between osteoblasts and osteoclasts playing a crucial role throughout the BM process (90–92). Osteoblasts promote bone formation by secreting bone morphogenetic proteins (BMPs) and endothelin-1 (ET-1) (93), while osteoclasts promote bone resorption through the RANKL and M-CSF signaling pathways (94). During PCBM, this balance is disrupted, leading to osteoblastic changes (characterized by new bone formation) and osteolytic changes (characterized by bone destruction visible on X-rays).

Specifically, PC cells secrete factors such as PTHrP and IL-11, which promote osteoclast differentiation and activity, thereby accelerating bone resorption (95–97). Simultaneously, PC cells can stimulate osteoblast activity by secreting ET-1 and BMPs, leading to bone sclerosis (98, 99). This imbalance between osteoblastic and osteoclastic processes not only alters bone structure but also provides a favorable environment for tumor cell growth. Studies have shown that regulating the activity of osteoblasts and osteoclasts can effectively control the progression of BM. For example, using RANKL inhibitors such as Denosumab significantly reduces osteoclast activity, thereby slowing bone resorption. Additionally, the application of ET-1 receptor antagonists can inhibit excessive osteoblast activity, reducing bone sclerosis (100, 101).

Notably, increasing evidence suggests that osteoblasts and osteoclasts play unique roles in the transition between dormancy and proliferation of disseminated tumor cells during BM (91). For instance, in multiple myeloma, the dormancy of cancer cells in the bone marrow is primarily associated with contact with osteoblasts, which initiate and maintain this state, while osteoclasts can disrupt this dormancy, reactivating cancer cells and promoting their proliferation (102). Similar phenomena have been observed in BM of breast and prostate cancers, although the underlying mechanisms remain unclear (91, 103). Limited studies indicate that osteoclasts and osteoblasts may directly interact with dormant tumor cells in the bone microenvironment or regulate the dormancy state of tumor cells near the endosteum by secreting various substances (104). Moreover, dormant tumor cells can recruit osteoclast progenitor cells, enhancing local osteoclast activity and disrupting tumor cell dormancy (105). This suggests that the “dormancy switch” of tumor cells is not only directly regulated by osteoclasts and osteoblasts but may also be influenced by factors secreted by tumor cells and other cells in their microenvironment. Dormant tumor cells are resistant to chemotherapy drugs targeting cell division and can evade immune surveillance (106), persisting as minimal residual disease (MRD). After achieving clinical remission and stopping treatment, these cells may be reactivated, leading to cancer recurrence. Therefore, future research should focus on the tumor microenvironment, particularly the external mechanisms triggered by the imbalance between osteoblasts and osteoclasts, to prevent intrinsic drug resistance and late biochemical recurrence of tumor cells. The 2020–2023 spike in “dormancy” related publications (**Figure 7A**) reflects growing recognition that overcoming therapeutic resistance requires precisely mapping these ecological interactions. Exploring new strategies to alter disease progression by modulating the bone marrow microenvironment is essential.

## 5 Limitation

In the present study, we have utilized a combination of bibliometric and bioinformatics analysis to explore PCBM.

While this approach has yielded significant insights, there are several limitations that warrant acknowledgment. Firstly, our study focused on English-language publications, potentially excluding valuable research from non-English speaking researchers, which could lead to a bias in the analysis. The concept of the Science Citation Index is based on Bradford’s Law of Bibliometrics, which can be used to define a set of core journals or publications. Journals included in the SCI-E database of WoS are described as the world’s leading journals due to their rigorous selection process (107). Therefore, the publications included in WoS can represent the representative research in this field. Secondly, there is a time lag between the publication of new papers after the retrieval date and the conduct of this study, and the retrieval information for 2024 is incomplete. Bibliometric trends can be influenced by recent publication biases, and our study may not fully represent historical perspectives or anticipate future shifts in research focus.

Moreover, our bioinformatics analysis was primarily based on the GSE32269 dataset from the GEO database. The reliance on a single data source may not capture the full spectrum of genetic variations and expressions in PCBM across diverse populations. Future studies should incorporate multiple datasets to enhance the representativeness and validity of the findings. Second, while GSE32269 remains a valuable resource for bone metastasis research, its age (published in 2011) raises questions about technological biases (e.g., microarray vs. RNA-seq). Newer datasets generated with advanced platforms (e.g., scRNA-seq) may capture transcriptional heterogeneity more comprehensively. However, GSE32269’s inclusion of normal bone marrow controls provides a critical baseline absent in many modern datasets. Future work should integrate multi-omics data to validate our findings.

Although our DEG analysis revealed key candidates like TMOD1, experimental validation (e.g., qPCR, immunohistochemistry) is needed to confirm their roles in PCBM pathogenesis. The lack of such validation may affect the reliability of these molecular findings. This limitation warrants further investigation to establish the biological significance of these molecular targets in PCBM pathogenesis.

## 6 Conclusion

The study integrates bibliometric analysis with bioinformatics to provide a comprehensive understanding of PCBM. In the realm of bibliometrics, the research meticulously examines the scientific literature to track the progression of research insights and identify key contributors. It reveals a notable increase in scholarly output over the past two decades, with the United States and China emerging as leading forces in this domain. The United States has consistently been at the forefront, with a substantial number of publications reflecting its robust research infrastructure and focus on innovative therapeutic strategies. Chinese contributions have been equally significant, indicating a rapid ascent in research capabilities and a growing emphasis on global health issues, especially for PCBM. Institutional collaborations between these two nations are particularly pronounced, suggesting a synergistic exchange of ideas and resources. The study’s bibliometric analysis also highlights the pivotal role of authorships in shaping the research narrative, with several researchers from both countries driving the scientific discourse through their influential publications.

While the study’s methodical approach yields valuable insights, it also acknowledges the limitations inherent in its methodology. The reliance on a single gene expression dataset and the necessity for experimental validation of computational findings are recognized. Future research endeavors should seek to corroborate these findings across multiple datasets and through laboratory experimentation. This concerted effort to bridge bibliometric trends with molecular profiles not only enhances the current understanding of PCBM but also sets the stage for more precise diagnostic and therapeutic strategies in the future.

## Data Availability

The original contributions presented in the study are included in the article/**Supplementary Material**, further inquiries can be directed to the corresponding authors.
