# Supplementary material for: BioBERT-powered synergy: advanced bibliometric and molecular insights into prostate cancer bone metastasis
Source: Front Immunol. 2025 Jun 18;16:1562559. doi: 10.3389/fimmu.2025.1562559 (PMC12213787; doi:10.3389/fimmu.2025.1562559)
Supplement: Supplementary file 4 [file DataSheet4.zip › Supplementary_Material.docx]

Supplementary Material

# 1 Supplementary Figures and Tables

## 1.1 Supplementary Figures

**Supplementary Figure 1.** **(A)** Countries or regions involved in the research on prostate cancer bone metastasis. **(B)** The collaborative network of countries/regions. Different nodes represent different countries or regions, and the links between the nodes indicate their collaborations and connections. The size of the nodes indicates the article volume in those countries/regions. **(C)** Overlay visualization of the countries analysis. The purple nodes represent keywords that appeared earlier, whereas the yellow nodes indicate keywords that have emerged more recently. **(D)** The top 10 most relevant affiliations in the field of prostate cancer bone metastasis research.

**Supplementary Figure 2. (A)** Author productivity through Lotka’s Law. **(B)** Top 10 authors’ production over time (2004–2024) on prostate cancer bone metastasis research. Ten authors were selected based on their productivity and citation frequency; most have published articles for at least 8 consecutive years, indicating their significant contributions to prostate cancer bone metastasis research.

**Supplementary Figure 3. (A)** Violin plot of the number of articles and reviews produced by authors. ***: P<0.001. The red violin plot represents articles, with the third quartile (Q3) at 11, the median (Q2) at 8, the first quartile (Q1) at 5, and the interquartile range (IQR) at 6. The yellow violin plot represents reviews (Q3=6, Q2=4, Q1=2, IQR=4). **(B)** Violin plot of the number of cited articles and reviews. ***: P<0.001. The red violin plot represents the number of cited articles (Q3=49, Q2=34, Q1=25, IQR=24), and the yellow violin plot represents the number of cited reviews (Q3=122.25, Q2=83, Q1=54, IQR=62.25).

**Supplementary Figure 4.** Overlay visualization of the keyword co-occurrence analysis. Purple nodes represent established keywords, while yellow nodes indicate recently emerging keywords.

**Supplementary Figure 5**. **(A)** Differentially expressed genes on each pathway of KEGG enrichment. **(B)** Differentially expressed genes on each pathway of biological process in GO enrichment. **(C)** Frequency of each gene in the abstract. **(D)** Frequency of each disease in the abstract.

## 1.2 Supplementary Tables

**Supplementary Table 1.** Top 10 authors with most number of articles.

| **RANK** | **AUTHOR** | **DOCUMENT** | **COUNTRY/REGION** | **INSTITUTION** |
| --- | --- | --- | --- | --- |
| 1 | Saad, Fred | 109 | Canada | Univ Montreal |
| 2 | Coleman, RE | 68 | England | Univ Sheffield |
| 3 | Pienta, Kenneth J | 51 | USA | Johns Hopkins Univ |
| 4 | Logothetis, Christopher J | 48 | USA | Univ Texas MD Anderson Canc Ctr |
| 5 | Chow, Edward | 47 | Canada | Univ Toronto |
| 6 | Lipton, Allan | 45 | USA | Penn State Univ |
| 7 | Fizazi, Karim | 44 | France | Univ Paris Saclay |
| 8 | Tombal, Bertrand | 43 | Belgium | Clin Univ St Luc |
| 9 | Smith, Matthew R | 43 | USA | Massachusetts Gen Hosp |
| 10 | Sartor, Oliver | 42 | USA | Tulane Canc Ctr |

**Supplementary Table 2.** Lotka’s Law statistics of papers written by authors on prostate cancer bone metastasis research from 2004 to 2024.

| Number of Articles | Number of Authors | Frequency |
| --- | --- | --- |
| 1 | 23830 | 0.792 |
| 2 | 3590 | 0.119 |
| 3 | 1177 | 0.039 |
| 4 | 524 | 0.017 |
| 5 | 312 | 0.010 |
| 6 | 168 | 0.006 |
| 7 | 119 | 0.004 |
| 8 | 90 | 0.003 |
| 9 | 54 | 0.002 |
| 10 | 42 | 0.001 |
| 11 | 28 | 0.001 |
| 12 | 27 | 0.001 |
| 13 | 18 | 0.001 |
| 14 | 15 | 0 |
| 15 | 12 | 0 |
| 16 | 9 | 0 |
| 17 | 7 | 0 |
| 18 | 5 | 0 |
| 19 | 9 | 0 |
| 20 | 7 | 0 |
| 21 | 7 | 0 |
| 22 | 4 | 0 |
| 23 | 6 | 0 |
| 24 | 3 | 0 |
| 25 | 3 | 0 |
| 26 | 4 | 0 |
| 27 | 2 | 0 |
| 28 | 2 | 0 |
| 29 | 2 | 0 |
| 32 | 1 | 0 |
| 33 | 2 | 0 |
| 36 | 1 | 0 |
| 37 | 4 | 0 |
| 38 | 1 | 0 |
| 40 | 2 | 0 |
| 44 | 2 | 0 |
| 48 | 1 | 0 |
| 95 | 1 | 0 |

**Supplementary Table 3.** Core Journals by Bradford’s Law.

| Zone | Number Of Journals | Percentage |
| --- | --- | --- |
| 1 | 31 | 3.0% |
| 2 | 124 | 12.0% |
| 3 | 880 | 85.0% |
| total | 1035 | 100.0% |

**Supplementary Table 4** Top 15 co-cited references related to prostate cancer bone metastasis.

| RANK | FIRST AUTHOR | SOURCE | ARTICLE TITLE | DOI | CITATION | DOCUMENT TYPE | YEAR |
| --- | --- | --- | --- | --- | --- | --- | --- |
| 1 | Parker C | New England Journal of Medicine | Alpha Emitter Radium-223 and Survival in Metastatic Prostate Cancer | https://doi.org/10.1056/NEJMoa1213755 | 2377 | Article | 2013 |
| 2 | Roodman GD | New England Journal of Medicine | Mechanisms of Bone Metastasis | https://doi.org/10.1056/NEJMra030831 | 1688 | Review | 2004 |
| 3 | Coleman RE | Clinical Cancer Research | Clinical Features of Metastatic Bone Disease and Risk of Skeletal Morbidity | https://doi.org/10.1158/1078-0432.CCR-06-0931 | 1681 | Review | 2006 |
| 4 | Fizazi Karim | Lancet | Denosumab versus zoledronic acid for treatment of bone metastases in men with castration-resistant prostate cancer: a randomised, double-blind study | https://doi.org/10.1016/S0140-6736(10)62344-6 | 1410 | Article | 2011 |
| 5 | Kwon ED | Lancet Oncology | Ipilimumab versus placebo after radiotherapy in patients with metastatic castration-resistant prostate cancer that had progressed after docetaxel chemotherapy (CA184-043): a multicentre, randomised, double-blind, phase 3 trial | https://doi.org/10.1016/S1470-2045(14)70189-5 | 1157 | Article | 2014 |
| 6 | Heidenreich A | European Urology | EAU guidelines on prostate cancer. Part II: Treatment of advanced, relapsing, and castration-resistant prostate cancer | https://doi.org/10.1016/j.eururo.2013.11.002 | 1097 | Review | 2014 |
| 7 | Stopeck AT | Journal of Clinical Oncology | Denosumab Compared With Zoledronic Acid for the Treatment of Bone Metastases in Patients With Advanced Breast Cancer: A Randomized, Double-Blind Study | https://doi.org/10.1200/JCO.2010.29.7101 | 1094 | Article | 2010 |
| 8 | Weilbaecher KN | Nature Reviews Cancer | Cancer to bone: a fatal attraction | https://doi.org/10.1038/nrc3055 | 921 | Review | 2011 |
| 9 | Saad Fred | Journal of the National Cancer Institute | Long-Term Efficacy of Zoledronic Acid for the Prevention of Skeletal Complications in Patients With Metastatic Hormone-Refractory Prostate Cancer | https://doi.org/10.1093/jnci/djh141 | 889 | Article | 2004 |
| 10 | Smith Matthew R | New England Journal of Medicine | Apalutamide Treatment and Metastasis-free Survival in Prostate Cancer | https://doi.org/10.1056/NEJMoa1715546 | 883 | Article | 2018 |
| 11 | Bamias A | Journal of Clinical Oncology | Osteonecrosis of the Jaw in Cancer After Treatment With Bisphosphonates: Incidence and Risk Factors | https://doi.org/10.1200/JCO.2005.02.8670 | 832 | Article | 2005 |
| 12 | Henry DH | Journal of Clinical Oncology | Randomized, Double-Blind Study of Denosumab Versus Zoledronic Acid in the Treatment of Bone Metastases in Patients With Advanced Cancer (Excluding Breast and Prostate Cancer) or Multiple Myeloma | https://doi.org/10.1200/JCO.2010.31.3304 | 828 | Article | 2011 |
| 13 | Even-Sapir | Journal of Nuclear Medicine | The detection of bone metastases in patients with high-risk prostate cancer: 99mTc-MDP planar bone scintigraphy, single- and multi-field-of-view SPECT, 18F-fluoride PET, and 18F-fluoride PET/CT | https://jnm.snmjournals.org/content/47/2/287 | 608 | Article | 2006 |
| 14 | Kirby M | International Journal of Clinical Practice | Characterising the castration-resistant prostate cancer population: a systematic review | https://doi.org/10.1111/j.1742-1241.2011.02799.x | 592 | Review | 2011 |
| 15 | Hess KR | Cancer | Metastatic patterns in adenocarcinoma | https://doi.org/10.1002/cncr.21778 | 592 | Article | 2006 |
